# High throughput calculations for a dataset of bilayer materials

**DOI:** 10.1038/s41597-023-02146-7

**Published:** 2023-04-21

**Authors:** Ranjan Kumar Barik, Lilia M. Woods

**Affiliations:** Department of Physics, University of South Florida, Tampa, Florida 33620 USA

**Keywords:** Two-dimensional materials, Electronic structure

## Abstract

Bilayer materials made of 2D monolayers are emerging as new systems creating diverse opportunities for basic research and applications in optoelectronics, thermoelectrics, and topological science among others. Herein, we present a computational bilayer materials dataset containing 760 structures with their structural, electronic, and transport properties. Different stacking patterns of each bilayer have been framed by analyzing their monolayer symmetries. Density functional theory calculations including van der Waals interactions are carried out for each stacking pattern to evaluate the corresponding ground states, which are correctly identified for experimentally synthesized transition metal dichalcogenides, graphene, boron nitride, and silicene. Binding energies and interlayer charge transfer are evaluated to analyze the interlayer coupling strength. Our dataset can be used for materials screening and data-assisted modeling for desired thermoelectric or optoelectronic applications.

## Background & Summary

Monolayered materials have attracted considerable research interest in science and technology due to their inherent novel properties compared to their bulk counterparts. Systems, such as transition metal dichalcogenides (TMDs)^1^, Janus monolayers^2^, MXene^3^, boron nitride (BN)^4^, phosphorene^5^, and others^6^, have proven as excellent candidates for thermoelectrics^7^, optoelectronics^8^, and spintronics^9^ nanoscale device applications. Starting with graphene^10^, layered materials and their heterostructures have been the source of emergent phenomena coupling their electronic, optical, thermal, and mechanical response with nontrivial topology. For example, hexagonal honeycomb materials can host valley Hall effect and quantum anomalous Hall effect providing a platform to study dissipation-less valleytronics^11^. Layered materials with broken inversion symmetry could lead to *k*-valley-dependent contrasted circular dichroism, enabling valley optical selection rules^12^. Different substrate environments can provide substantial Rashba effect, making it possible to design spintronics devices such as spin-field effect transistors^13^.

Layered materials are typically composed of chemically inert monolayers that can be organized to make various multilayer composites. In addition to individual layers, systems that consist of finite number of identical monolayers or heterostructures made of layers with different chemical compositions give rise to new subfields in science and technology. The diversity is practically limitless given the ever increasing library of layered materials^14,15^. Recent large scale simulations predict over 4000 2D materials with different chemical compositions and lattice space groups^16^. Some of them have been synthesized such as transition metal dichalcogenides (TMDs)^17^, hexagonal boron nitride (hBN)^18^, graphene^19^, MSe_2_ (M = Zr, Hf)^20,21^, ZrS_2_^22^, TiS_2_^23^, MoSSe^24^ and so on, while others are waiting to be realized in the laboratory.

In addition to their monolayer phase, bilayers constitute a separate subset of 2D materials whose different stacking patterns and interlayer interactions become additional sources of new physics^25,26^. In particular, the semimetallic behavior of graphene limits its application in the next-generation device application; however, its bilayer has a tunable band gap via external electric fields, enabling its device applications^27^. On the other hand, various types of stacking patterns in bilayers could offer a platform to study different novel phenomena, including magneto-electric coupled multiferroics effect^28^, different optical response^29,30^, topology^31^, magnetism^32^, and Rashba spin-splitting^33^. Besides, different twisting angles of monolayers can exhibit moiré patterns resulting in many intriguing properties, such as Mott insulating behavior^34^, topological superconducting state^35^, tunable band gaps^36^, and light-matter interactions^37^ providing an opportunity to study twistronics in a large class of bilayers.

In this study, we report high throughput Density Functional Theory (DFT) calculations of van der Waals bilayer materials, composed of chemically inert dynamically stable nonmagnetic monolayers as reported in the C2DB database site^16^. We consider monolayers with hexagonal and square type lattices with various stackings obtained by rotating or sliding the top layer against the bottom layer as dictated by the symmetry of each monolayer. Besides, many of the different stacking patterns can be viewed as variations of the AA and AB bilayer orientations for bilayer graphene^38^. For each of the constructed 760 bilayers, the energetically most stable configuration is reported together with several electronic and transport properties. By calculating the density of states (DOS) and band structures, energy gaps in the 0≤*E*_*g*_ < 6 eV are found, further highlighting the role of spin orbit coupling (SOC). Lattice parameters, interlayer separations, van der Waals coupling, and charge transfer are also calculated to generate data driven information of interlayer effects in bilayers. Additionally, calculated Seebeck coefficient, electrical conductivity, and effective band structure masses provide the foundation for a transport database^39^ properties of 2D materials. The reported data gives valuable information for screening and identifying materials with targeted properties as well as a set of descriptors to be used in future statistical models based on data mining and machine learning methods^40,41^.

## Methods

### Work flow

**Fig. 1:**
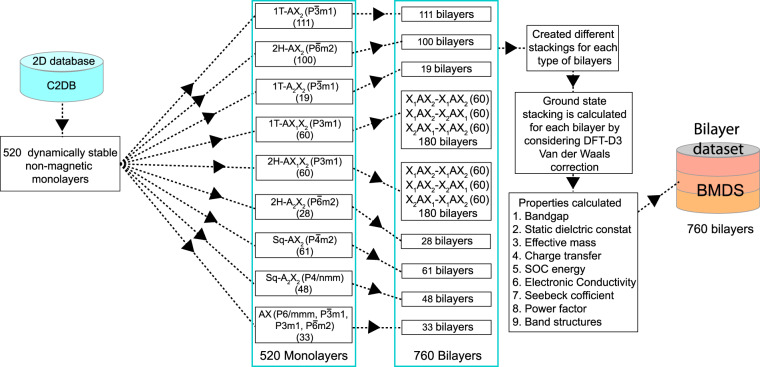
High through-put work flow for building the bilayer materials dataset (BMDS), where the symmetries of 520 dynamically stable nonmagnetic 2D structures are analyzed to construct and simulate bilayers made of identical monolayers using the DFT methods.

The high throughput computational work flow to build the bilayer materials dataset is shown in Fig. 1. The initial set of materials is taken from the C2DB database^42^, where we consider non-magnetic and dynamically stable monolayers for further screening. The breakdown of the stoichiometry, group symmetry, and the corresponding number of monolayers are also given explicitly. A total of 520 monolayers have been examined and schematic representation of those monolayers is provided in Fig. S1 with their lattice constants given in Fig. S2 of the Supporting Information.

The symmetries in each monolayer type have been analyzed to generate different bilayer stacking patterns having chemically identical monolayers. These stacking patterns are created by considering an initial interlayer separation of 3.3 Å. Besides the atomic registry dependence, the different surface atoms in the Janus (AX_1_X_2_) 1 T and 2H phases have provided additional adaptability for constructing the Janus-derived bilayers. We framed three distinct bilayers phases as X_1_AX_2_-X_2_AX_1_ (60 structures), X_1_AX_2_-X_1_AX_2_ (60 structures), and X_2_AX_1_-X_1_AX_2_ (60 structures) with X_2_-X_2_, X_2_-X_1_, and X_1_-X_1_ atoms making up the interface region. Accordingly, 760 bilayers with nine different phases have been formulated from the initial 520 monolayers.

DFT self-consisted calculations are then performed by considering the van der Waals interactions to obtain the relaxed structures of all bilayers. Energies of the stackings in each bilayer type are compared, and the stacking order of the energetically most stable system is identified as the ground state configuration for our bilayer materials dataset (BMDS). Subsequently, electronic structure calculations are carried out to obtain the energy band gaps with and without SOC, the magnitude of the SOC energy, charge transfer between the layers, effective masses at the valence band maxima and conduction band minima of the most stable bilayers. These results are reported in the BMDS. Transport properties, such as the scaled electrical conductivity (*σ*/*τ*), Seebeck coefficient (*S*), and scaled Power factor (*S*^2^*σ*/*τ*) within a constant relaxation time approximations *τ* are also calculated. These data are also provided in our BMDS dataset^43^.

Detailed computational calculations and reported materials properties are discussed in what follows.

### Materials design and density functional theory calculations

The construction of the differently stacked bilayers is facilitated by analyzing the crystal symmetries of the constituent monolayers^44–50^, which is implemented by using combinations of different slidings and/or 180° rotations of the hexagons or squares making up the monolayer lattices. The atomic representation of the stacking patterns with a convenient notation of each class of materials is given in Figs. S3–S12 in the Supporting Information. The centrosymmetric A*X*_2_ and A_2_*X*_2_ hexagonal systems with three and four atoms in the unit cell and the non-centrosymmetric Janus monolayers A*X*_1_X_2_ have six possible bilayer stacking patterns. The square monolayers in A*X*_2_ and A_2_*X*_2_ stoichiometries with three or four unit cells have four distinct bilayer stacking patterns. The configurations of monolayers with two atoms per unit cell depend upon the presence of finite thickness, such that planar materials (such as graphene) have two stacking patterns while finite thick materials have four possible stacking patterns.

The stacking configurations for the considered bilayers are calculated using DFT methods as implemented in the Vienna Ab initio Simulation Package (VASP)^51^. The projector-augmented wave (PAW) potentials are used to incorporate the ion-electron interactions in the calculations^52^. The Perdew-Burke-Ernzerhof (PBE) functional within the generalized gradient approximation is adopted to estimate the exchange-correlation potential^53^. To expand the wavefunctions on a plane-wave basis, a kinetic energy cutoff of 600 eV is used with an energy convergence threshold of 10^−6^ eV. The structures were relaxed by utilizing a conjugate gradient scheme until each component of Hellmann-Feynman forces on the atoms was less than 0.005 eVÅ^−1^. A Γ-centered 12 × 12 × 1 Monkhorst-Pack^54^ k-point grid is considered for sampling the Brillouin Zone. A minimum vacuum layer of 15 Å is taken to avoid interactions with adjacent layers. Electronic band structure and transport properties are calculated by considering the SOC effect. The charge difference between the monolayer and bilayer has been calculated by Bader charge analysis^55^. The van der Waals interaction in different stacking is approximated by considering the DFT-D3 method with a zero-damping function^56^. Effective masses of VBM and CBM are calculated using the SUMO PYTHON package^57^.

### Structural, electronic, and dielectric properties of bilayer materials

**Fig. 2:**
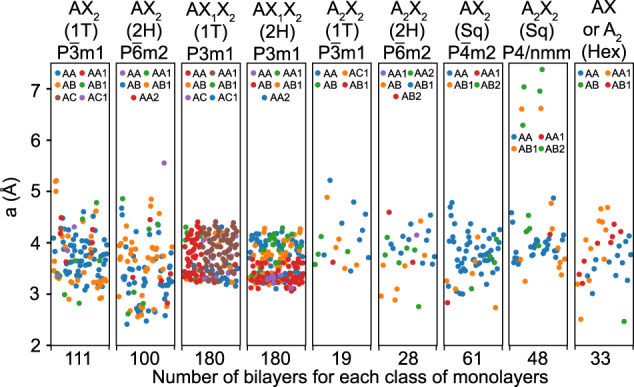
Lattice parameters for bilayers formed from different classes of monolayers. The scattered plots contain the data for most energetically stable stacking order for each material represented by a distinct color dot.

**Fig. 3:**
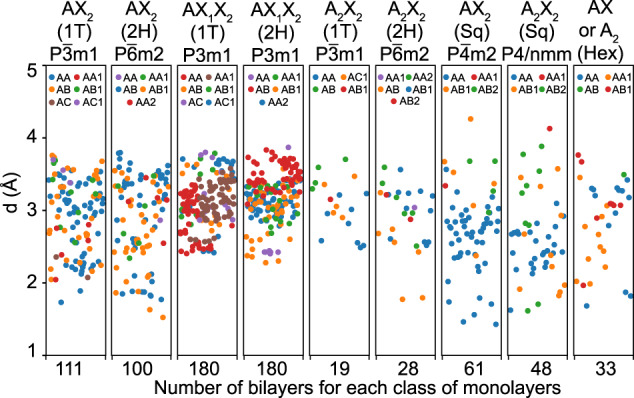
Interlayer distances for bilayers formed from different classes of monolayers. The scattered plots give data for the most energetically stable stacking order for each material. The calculations include van der Waals corrections.

All the data presented here concerns the ground state for each bilayer, which is identified by comparing the total energies as found from DFT and selecting the minimum value

1 $$E=min\left\{{E}_{1},{E}_{2},\ldots ,{E}_{n}\right\}$$

where *E*_1_, *E*_2_… *E*_*n*_ are the total DFT energies for all *n* possible stacking patterns of a given bilayer, as explicitly given in Figs. S3–S12 in the Supporting Information. Scattered plots of the lattice constant *a* and interlayer separation *d* in terms of number of materials for all nine bilayer sub-classes are shown in Figs. 2, 3, respectively, where each ground state is represented by a distinct color dot. The stoichiometry and space group belonging to their respective monolayer constituents are also denoted. In the CSV spreadsheet, we show a full list of both lattice constants *a* and *d*, which can further be compared with the lattice constants *a* of the monolayers given in Fig. S2 in the Supporting Information. Although many bilayers are found to be anisotropic, the difference between the *a* and *b* lattice constants is rather small. In most bilayers, *a* lies within 3–4.5 Å, and this trend is especially pronounced for the 1 T and 2 H Janus (AX_1_X_2_) materials where all data is concentrated in that region. We find that six materials, K_2_Br_2_, K_2_Cl_2_, K_2_I_2_, Rb_2_Br_2_, Rb_2_Cl_2_, and Rb_2_I_2_, in the square lattice phase A_2_X_2_ (P4/nmm), have much larger bilayer lattice parameters which is consistent with the data for the corresponding monolayers (monolayer lattice parameters are mentioned in Figure S2 in the Supplementary Information).

The numerical data for the interlayer distance *d* as obtained from DFT is also given in the CSV spreadsheet. For the large majority of systems *d* > 2.65 Å, which implies that chemical effects are relatively small and the bilayer may be considered as van der Waals systems. Nevertheless, several bilayers, especially some that are formed by hexagonal and square AX_2_ and square A_2_X_2_ monolayers have *d* < 2.65 Å indicating that stronger interlayer interactions might be present.

**Fig. 4:**
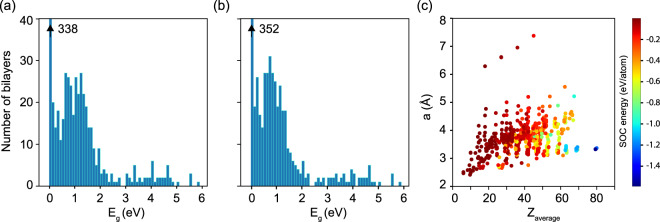
Histogram plot of the band gaps of all bilayers showing number of materials without (**a**) and with (**b**) the SOC effect; (**c**) scattered plot of the lattice parameter as a function the average atomic number (${Z}_{average}=\frac{{\sum }_{i=1}^{N}{n}_{i}{Z}_{i}}{{\sum }_{i=1}^{N}{n}_{i}}$, where n_*i*_ is the number of ith atom having atomic number *Z*_*i*_ present in the unit cell and *N* is total number of atomic species) of the bilayers. The dot color (color bar shown to the right of the panel) corresponds to the strength of the SOC energy per atom. All data are for the most stable bilayers configurations with included van der Waals interactions.

After the energetically most stable bilayer configuration for each bilayer is identified based on Eq. 1, the DFT electronic band structure is calculated. The energy band gaps *E*_*g*_ are extracted from these results and grouped in a 0.1 eV intervals for each bilayer type. The data is then plotted in Fig. 4 by a histogram representation for the data without (panel (a)) and with SOC (panel (b)). Since the calculations are done with standard semi-local functionals, which frequently underestimate the energy gaps, it is possible that for some materials *E*_*g*_ may differ from the ones given here. Nevertheless, our results are consistent with the level of DFT calculations in the monolayer C2DB database^16,42^. They can also provide a good idea about the metallic-semiconducting-insulating properties of bilayer materials, which could be especially valuable for initial screenings of materials with desired properties. Figure 4 shows that many bilayers are either metals (338 structures) or small gap semiconductors (about 100 structures). Besides, 49 bilayers have *E*_*g*_ > 3 eV, indicating that such nano-structured insulators make up a smaller portion in the pool of studied materials. The inclusion of SOC results in an overall decrease of *E*_*g*_, however, the general trend of most materials being metals or small gap semiconductors is preserved, as seen in Fig. 4b. The band gap values are found to be consistent with the ones reported for 3D weakly bound layered solids^15^, where the majority of the materials are either metals or small gap semiconductors.

From the calculations we further show the correlation between the lattice parameters, average atomic numbers, and strength of SOC of the considered bilayers, given in the scattered plot in Fig. 4c for the ground states of the bilayers. Given that most topologically nontrivial materials have large SOC effects^58,59^, the bilayers from our dataset with SOC > 0.4 eV maybe good candidates for further screening in terms of their topological characteristics.

**Fig. 5:**
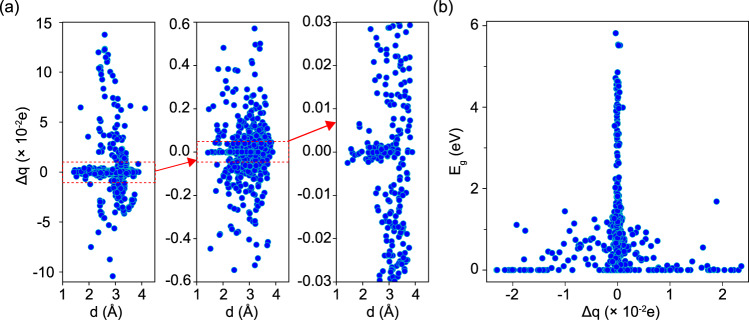
(**a**) Charge difference Δ*q* between an individual monolayer and the monolayer as part of the bilayer as a function of interlayer distances and (**b**) band gap E_*g*_ as a function of charge difference for the energetically most stable systems. The van der Waals correction is included in all calculations.

Another important property for the interlayer interaction is the charge transfer that occurs upon bringing the two monolayers together. Using Bader charge analysis, here we calculate the charge difference Δ*q* between the total charge of the individual monolayer (subscript ML) and the total charge of the monolayer as part of the bilayer structure (subscript sublayer in BL) using the relation,

2 $$\Delta q=\mathop{\sum }\limits_{i}^{N}[{q}_{i}{M}_{i}{| }_{ML}-{q}_{i}{M}_{i}{| }_{sublayerinBL}],$$

where *q*_*i*_ is the charge of i-th atom (*M*_*i*_) and the summation runs over the total number of atomic species in each monolayer class. This is a quantitative measure of the charge redistribution occurring in the bilayer formation as the monolayers are brought together. Data for Δ*q* as a function of the interlayer separation is given in Fig. 5a as a scattered plot for the energetically most stable bilayers. The negative and positive spreads of Δ*q* in Fig. 5a show charge transfer that occurs to and from the monolayer upon forming the bilayer. The results suggest that for the majority of materials, the charge transfer is rather small lying in the −0.5 × 10^−2^e < Δq < 0.5 × 10^−2^e range. This is consistent with a weaker interlayer coupling, also discussed earlier in the context of the interlayer separation. Further information can be obtained from the *E*_*g*_ vs Δ*q* correlation displayed in Fig. 5b. It is found that there is a Gaussian-like spread of the gap with Δ*q* for bilayers with *E*_*g*_ ≠ 0, while in metallic systems charge transfer occurs in the entire studied range.

**Fig. 6:**
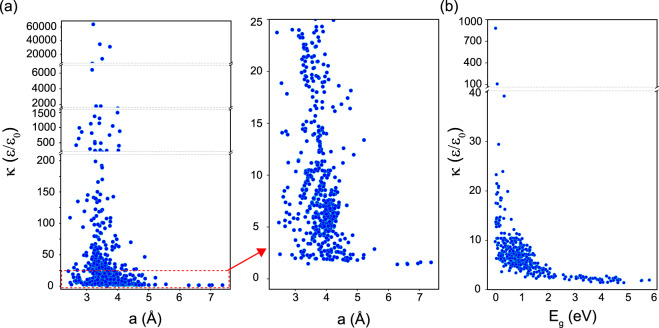
Scattered plot of the dielectric constant scaled by the vacuum dielectric constant *ε*_0_ as a function of the lattice constant (**a**) and and energy band gap (**b**) of the bilayer materials.

The dielectric constant *κ* is also calculated within DFT, and results for *κ* as a function of the lattice constant are shown in Fig. 6a. The zoom-in view of the *κ* < 25 window shows the distribution of the data for bilayers with smaller dielectric constant. It is found that about 541 bilayers have *κ* < 25, while 60 systems have *κ* > 100. Figure 6b further shows the dielectric constant vs the energy band gap of the bilayers in their ground state. This data may be useful for the search of “high-*κ*” and/or large gap dielectric nano-structured materials for the next generation electronic devices^60,61^. In addition to large *κ* responsible for enhanced capacitive coupling, dielectrics with a large band gap are needed to prevent leakage currents between electrodes for successful integration.

### Transport calculations

Transport properties such as electrical conductivity, Seebeck coefficient, and power factor are widely used for evaluating the materials thermoelectric figure of merit *ZT* = *S*^2^*σ*T/*κ*_*th*_, where *S*, *σ*, *T*, and *κ*_*th*_ are the Seebeck coefficient, electrical conductivity, temperature, and thermal conductivity, respectively. Materials with higher ZT are sought out for efficient thermoelectric devices. One way to achieve this goal is to look for materials with large values of their power factors *S*^2^*σ*. Using the semi-classical linearized Boltzmann transport theory within the constant relaxation time (*τ*) approximation (*τ* is material dependent and it typically requires separate experimental and theoretical studies) as implemented in the BoltzTraP2 code^62^, we have calculated *S*, *σ*, and *S*^2^*σ* for the energetically most stable bilayers. The simulations are performed with 3240 *k*-points, which is a dense enough mesh to obtain the transport properties.

**Fig. 7:**
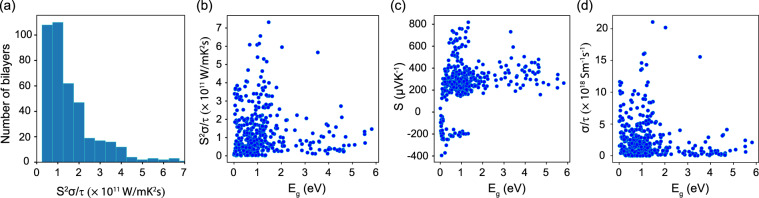
(**a**) Histogram plot for the number of materials and their power factors (*S*^2^*σ*/*τ*), (**b**) the power factor *S*^2^*σ*/*τ* as a function of *E*_*g*_ for bilayers with non-zero band gap at chemical potential *μ* = 0 and *T* = 300 K, (**c**) the Seebeck coefficient *S*, and (**d**) conductivity (*σ*/*τ*) as a function of *E*_*g*_ for bilayers with non-zero band gap at chemical potential *μ* = 0 and *T* = 300 K.

**Fig. 8:**
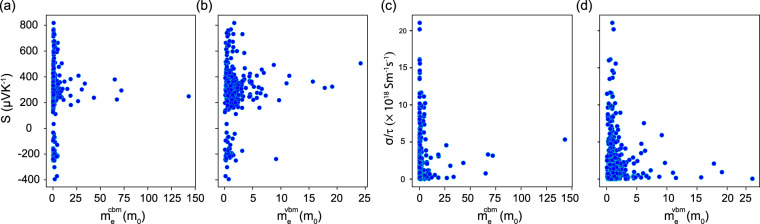
Scattered plot of Seebeck coefficient vs: (**a**) the effective mass at the conduction band minimum; (**b**) the effective mass at the valence band maximum. Scattered plot of the scaled by the constant relaxation time electrical conductivity vs: (**a**) the effective mass at the conduction band minimum; (**b**) the effective mass at the valence band maximum. All masses are given in terms of the electron rest mass *m*_0_. The reported data correspond to zero chemical potential and 300 K temperature.

After calculating *S* and *σ*/*τ*, we obtain the rescaled by the relaxation time power factor. A histogram plot distribution of *S*^2^*σ*/*τ* for the number of considered bilayers is shown in Fig. 7a. The correlation between the power factor correlates and the band gap of the bilayers is also shown in Fig. 7b. The bilayers with large power factors may be a starting point to search for materials with improved thermoelectric properties at the nanoscale. The effective mass associated with each electronic structure is an important descriptor for the materials power factors and figures of merit as reported in high throughput calculations for bulk materials (mostly binary structures)^63,64^. These quantities are readily available from DFT calculations, which can help one to assess if a given material may be a potential thermoelectric candidate. In Fig. 8, we give *S* and *σ*/*τ* values for bilayers with nonzero *E*_*g*_. One observes that largest *σ*/*τ* and S values are found for materials with smaller m_*e*_ and m_*h*_, which correlates well with data for 3D bulk materials^63,64^.

## Data Records

Our BMDS dataset containing 760 bilayer materials are composed of identical dynamically stable and nonmagnetic monolayers from the C2DB. The reported data contains information for the ground state stacking order of each material. All calculated data for the ground state bilayers are given in a CSV spreadsheet file and it can be found on figshare^43^. The figures for the electronic band structure with considering relativistic SOC effect and the transport coefficients at different temperature with varying the chemical potential are also provided in figshare. The description of the columns in the spreadsheet are shown explicitly the Table 1. We have provided two columns labeled as “materials” and “bilayer_materials” in the CSV file, which correspond to the monolayer material name id from C2DB and our BMDS bilayer materials name and its id, respectively. We have included lattice constants of monolayers and bilayers, interlayer distances, DFT total energies, binding energies, unit cell surface areas, space groups, band gaps with and without considering the SOC effect, dielectric constants, SOC energies, and charge transfer (taking the bottom layer as a reference) data in the CSV file.

**Table 1:** Detailed description of column names in the CSV spreadsheet containing the BMDS dataset.

Columns names	Type	Unit	Descriptions
materials	string	None	Monolayer materials name and id from C2DB database
bilayer_materials	string	None	Our BMDS bilayer materials name and its id
ground_state_stacking	string	None	Energetically most stable bilayer stacking configuration
a_mono	float	Å	Lattice parameters of monolayers
a	float	Å	Lattice parameter *a* of relaxed ground state stacking bilayer
b	float	Å	Lattice parameter *b* of relaxed ground state stacking bilayer
DFT_energy_bi	float	eV	DFT energy of relaxed ground state stacking bilayer
magnetic_moment	float	*μ*_*B*_	Magnetic moment of each bilayer
formation_energy	float	eV/atom	Formation energies of bilayers
area	float	Å^2^	Surface area of unit cell of relaxed ground state stacking bilayer
d	float	Å	Interlayer distance of relaxed ground state stacking bilayer
space_group	string	None	Space group of relaxed ground state stacking bilayer
no_soc_bandgap	float	eV	Band gap of relaxed ground state stacking bilayer without SOC
soc_bandgap	float	eV	Band gap of relaxed ground state stacking bilayer with SOC
dielectric_constant	float	None	Static dielectric constant of relaxed ground state stacking bilayer
soc_energy	float	eV/atom	SOC energy per atom
del_e_bilayer_bottom_and_mono	float	e	Charge transfer from bottom layer of bilayer
PF_xx	float	W/mK^2^s	Power factor along in-plane direction
PF_zz	float	W/mK^2^s	Power factor along out of plane direction
S_xx	float	*μ*VK^−1^	Seebeck coefficient along in-plane direction
S_zz	float	*μ*VK^−1^	Seebeck coefficient along out of plane direction
*σ*_xx	float	Sm^−1^s^−1^	Conductivity along in-plane direction
*σ*_zz	float	Sm^−1^s^−1^	Conductivity along out of plane direction
effective_mass_CBM	float	m_0_	Effective mass at conduction band minimum
effective_mass_VBM	float	m_0_	Effective mass at valence band maximum

All properties are calculated for relaxed stable stacking bilayers.

Transport properties, such a Seebeck coefficient, power factor, and conductivity at T = 300 K and *μ* = 0 for insulating bilayers are also reported in the CSV file. Additionally, effective masses at the VBM and CBM for insulating materials are included in the CSV file. Transport properties and effective masses of VBM and CBM are obtained for bilayers with nonzero energy gap. For metallic systems, that block is denoted as “metal”. Note that we considered all reported dynamically stable and nonmagnetic C2DB monolayers, however, some bilayers did not attain a stable interlayer separation, which became quite large or very small during the relaxation process (>8 Å or <1 Å in most cases). Such bilayers are discarded and the reported 760 bilayers are well converged structures. Also, while we considered only nonmagnetic monolayers, 24 bilayers acquired finite magnetic moments (>0.5 *μ*_*B*_). Therefore we have added a column for magnetic moment in the CSV file. For these magnetic bilayers, we have reported the spin polarized calculations. We find that all magnetic bilayers are metallic, thus their transport properties are not evaluated.

## Technical Validation

For the bilayer dataset presented here, we have taken electronically and dynamically stable nonmagnetic monolayers from the well known C2DB database (https://cmrdb.fysik.dtu.dk/c2db/cmrdb-c2db). First principles calculations are done using DFT methods with included van der Waals interactions. Such an approach is extensively used by the solid state physics and chemistry communities for materials predictions and property investigations. VASP is a premier quantum mechanical software package through which many emerging databases (including C2DB) are built. Each step of our high throughput calculations is checked with stringent convergence criteria. The stacking order of each bilayer is generated by utilizing previously established symmetry based criteria capturing the energetically most stable pattern configurations as reported by numerous references^44–50^.

**Fig. 9:**
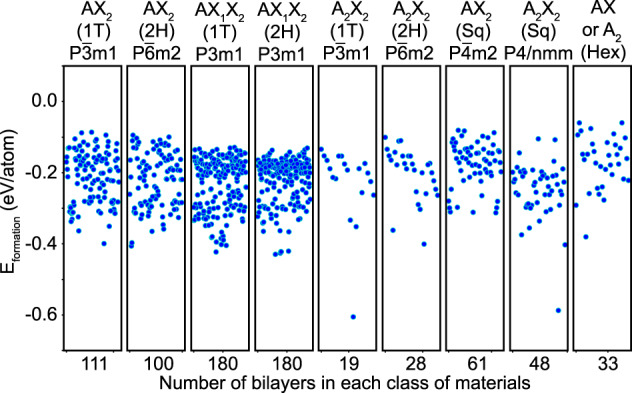
Scattered plot of the formation energy for the most stable configuration as a function of number bilayer materials in each bilayer class. Negative *E*_*formation*_ is indicative of the structural stability of the bilayers.

The vast majority of bilayer materials in BMDS have not been studied before. Therefore, we examine their stability by calculating the bilayer formation energy as: *E*_*formation*_ = *E*_*bilayer*_−2 × *E*_*monolayer*_, where *E*_*monolayer*_ and *E*_*bilayer*_ are the total energies of monolayer and its corresponding ground state stacked bilayer structures, respectively. Normally, negative formation energy values indicate structural stability of the bilayer. Figure 9 shows *E*_*formation*_ for the studied materials. All bilayers are found to have negative formation energies, such that −0.7 eV/atom < *E*_*formation*_ < 0 eV/atom. The only exception is the bilayer MgCl_2_ in the square AX_2_ phase, for which *E*_*formation*_ = 1.24 eV/atom and it is not explicitly given in Fig. 9. We also note that all individual monolayers in our bilayer dataset are reported as dynamically stable in the C2DB database^16,42^ due to the lack of negative phonon modes. Additionally, several studies have shown that many bilayers composed of monolayers from the AX_2_, AX_1_X_2_, AX and A_2_ classes also have phonon band structure lacking negative modes^65–72^ demonstrating a dynamical stability for stacking patterns considered here as well. Although the phonon dispersion is an important factor for the stability of materials, it requires careful examination in terms of type and size of the constructed supercells, different functionals, and/or smearing parameters^73–77^. While small differences in bond lengths and angles may not show noticeable differences in the electronic structure, they are detrimental for the phonon band structure. Changes in the interlayer separation, which is closely related to the particular vdW dispersion method, can also have important consequences for the presence or lack of negative phonons of layered materials. We note that the monolayers dynamical stability and the several studies on vdW bilayers are strong indicators for the stability of the bilayer dataset here. The crucial influence of the specific DFT approximations and parameters on the phonon dispersion is materials dependent, thus phonon calculations are not part of this high throughput study.

The electronic structure properties of several bilayers have also been studied computationally by others. A comparison of the interlayer distances, band gaps, and stacking pattens of previously reported and calculated here data are shown in Table 2. Most of the stacking patterns are identical to our results (indicated with (a) label), with the exception of what was reported for the Janus bilayers made of TeMoSe and SeMoTe monolayers (indicated with a (b) label), mainly due to the more stringent relaxation conversion criteria used in our simulations. Table 2 further shows that there are greater dissimilarities in some band gaps (although good agreement is found for many cases). The main reason is that a variety of exchange-correlation functionals and van der Waals interaction approximations are considered by the different authors. Nevertheless, there is generally a good agreement for the interlayer distances, especially for most transition metal dichalcogenides^78^ for which *d* = 3–3.5 Å are found.

**Table 2:** A comparison of interlayer distances, band gaps, and stacking pattens of several bilayers in the BMDS dataset with results reported in other references.

Bilayer materials and id	d (Å) (BMDS)	d (Å) (previously reported)	E_*g*_ (eV) (BMDS)	E_*g*_ (eV) (previously reported)	Stacking
SeHfS-SeHfS	3.086	2.802	0.715	0.415	AA (a)^79^
SeHfS-SHfSe	3.086	2.8	0.703	0.426	AA1 (a)^79^
SHfSe-SeHfS	3.219	2.868	0.736	0.736	AA1 (a)^79^
TeMoSe-TeMoSe	3.384	3.19	0.836	0.562	AB (b)^33^
SeMoS-SeMoS	3.153	3.12	0.9419	0.96	AA2 (a)^80,81^
TeMoS-TeMoS	3.153	3.018	0.1246	0	AA2 (a)^33^
SeWS-SeWS	3.197	—	1.1573	1	AB (a)^82^
TeMoSe-SeMoTe	3.093	3.01	0.6275	0.46	AA2 (a)^33^
SeMoS-SMoSe	3.022	2.831	0.9418	0.81	AA2 (a)^81^
SeWS-SWSe	3.048	—	1.1566	1.0	AA2 (a)^82^
SeMoTe-TeMoSe	3.436	3.289	1.0617	1.001	AB (b)^33^
SMoSe-SeMoS	3.348	3.2	1.3295	1.23	AB (a)^83^
SWSe-SeWS	3.254	—	1.2952	1.3	AB (a)^82^
HfSe_2_-HfSe_2_	3.114	2.96	0.5143	1.07	AA (a)^65^
PtS_2_-PtS_2_	2.253	2.54	0.7483	0.99	AA (a)^84^
PtSe_2_-PtSe_2_	2.274	2.14	0.1876	0.19	AA (a)^85^
PtTe_2_-PtTe_2_	2.40	2.39	0	0	AA (a)^86^
SnS_2_-SnS_2_	3.029	2.95	1.51	1.53	AA (a)^49^
ZrS_2_-ZrS_2_	2.987	—	1.079	1.09	AA (a)^87^
ZrSe_2_-ZrSe_2_	3.095	3.08	0.3796	0.99	AA (a)^65^
MoS_2_-MoS_2_	3.072	—	1.31	1.24	AB (a)^88,89^
MoSe_2_-MoSe_2_	3.206	—	1.215	1.2	AB (a)^88,89^
MoTe_2_-MoTe_2_	3.427	3.41	0.980	0.98	AB (a)^89,90^
NbSe_2_-NbSe_2_	2.939	3.042	0	0	AB1 (a)^91^
WO_2_-WO_2_	2.547	—	1.2	1.22	AA2 (a)^89^
WS_2_-WS_2_	3.161	—	1.507	1.43	AB (a)^89^
WSe_2_-WSe_2_	3.218	—	1.389	1.23	AB (a)^89^
WTe_2_-WTe_2_	3.43	—	1.024	0.99	AB (a)^89^
BN-BN	3.453	3.34	4.517	4.56	AA1 (a)^92^
GaN-GaN	2.330	2.47	2.02	2.01	AA1 (a)^93^
As_2_-As_2_	3.270	3.270	0.920	0.7	AA (a)^94^
C_2_-C_2_	3.493	3.46	0	0	AB (a)^95^

The stacking pattern notation is used as per the BMDS conventions. The label a (b) denotes bilayers whose stacking pattern for the energetically most stable configuration obtained here and by others is the same (different). In some cases, values for *d* and/or *E*_*g*_ are not reported.

## Usage Notes

The data for the bilayers BMDS can be accessed directly from the CSV spreadsheet provided in figshare^43^. The dataset can further be expanded by considering bilayers formed of other 2D monolayers with different lattice structures which may or may not host magnetic states.

## Supplementary information

Supplementary Information: High throughput calculations for a dataset of bilayer materials

## Data Availability

In addition to the CSV file, several components to work with the reported bilayer materials can be found in figshare^43^ and in GitHub (https://github.com/rkb12/BMDB-databaseBMDB-database). In the stacking-pattern-PYTHON-code-for-each-class zipped directory, a code that obtains all possible stacking patterns (following the naming notation in Fig. 2) are given. Inside each subdirectory, a prototype example of monolayer structure and the corresponding stacking code are given. Executing the code in the same directory will create the stacking pattens for the respective class of bilayer. In the zipped soc-bandstructure-code, an in-house code generating the band structure with SOC is provided. The code reads a VASP output file and generates a graphical representation for the band structure. In the zipped transport-cal-code, an in-house code generating the transport properties such as conductivity (*σ*/*τ*), Seebeck coefficient (S), and power factor (S^2^*σ*/*τ*) as function of chemical potential at different temperature for each bilayer material. The code reads VASP and BoltzTraP output files and generates a graphical representation for the transport properties. In the zipped soc-bandstructure-figures and transport-figures a graphical representation for the energy band structure (with and without SOC) and transport properties for the ground state stacking compositions of all bilayer materials are also given.
