# Supplementary material for: High throughput calculations for a dataset of bilayer materials
Source: Sci Data. 2023 Apr 21;10:232. doi: 10.1038/s41597-023-02146-7 (PMC10121719; doi:10.1038/s41597-023-02146-7)
Supplement: Supplementary file 1 — Supplementary Information: High throughput calculations for a dataset of bilayer materials [file 41597_2023_2146_MOESM1_ESM.pdf]

Supplementary Information: High throughput calculations for a dataset of bilayer materials

Ranjan Kumar Barik[1] and Lilia M Woods[1]

[1] *Department of Physics, University of South Florida, Tampa, FL 33620, United States*[*]

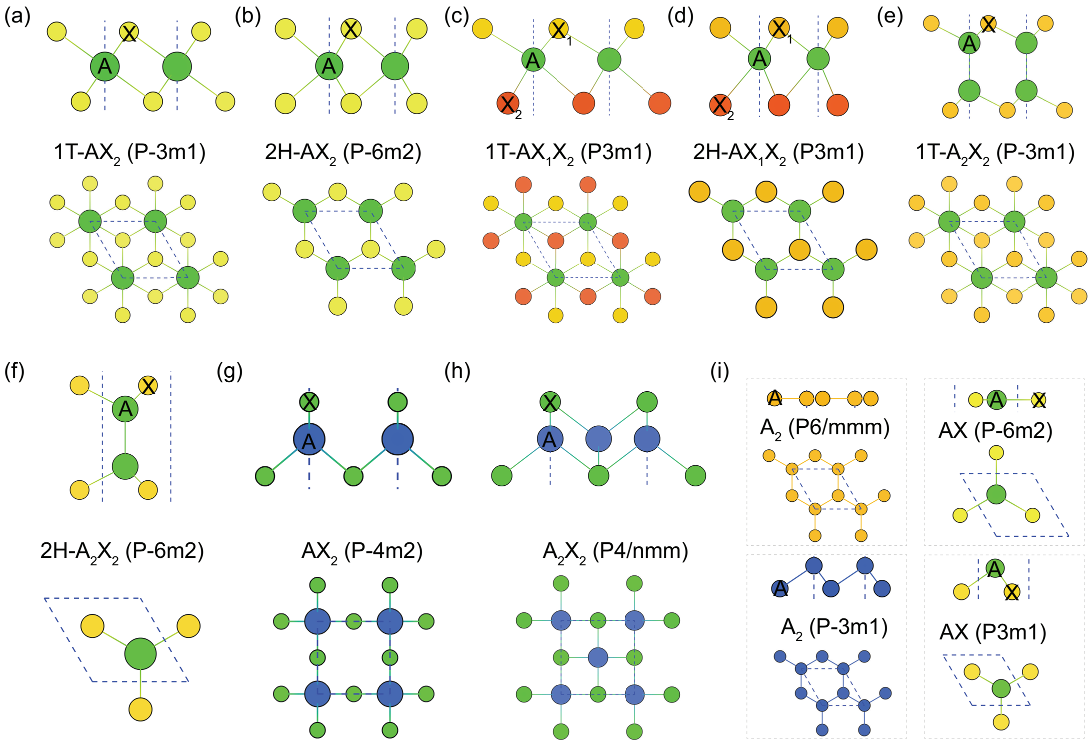

Figure S1: Monolayers from different symmetry classes taken from the C2DB database to construct the bilayer materials database BMDB. The side and top views for each monolayer are shown in the top and bottom images, respectively, for each panel. The cation and chalcogen atoms are denoted as A and X, respectively, in each panel.

The bilayer stacking configurations for the 2H-AX_1X_2 Janus monolayers are not given explictly. They are obtained in the same manner as the ones for the 2H-AX_2 bilayers in Figure S4. The different atomic combinations making up the interface region give rise to three distinct patterns, similar to Figure S5, S6, and S7 for 1T-AX_1X_2 Janus bilayers.

* sran9125@; lmwoods@usf.edu

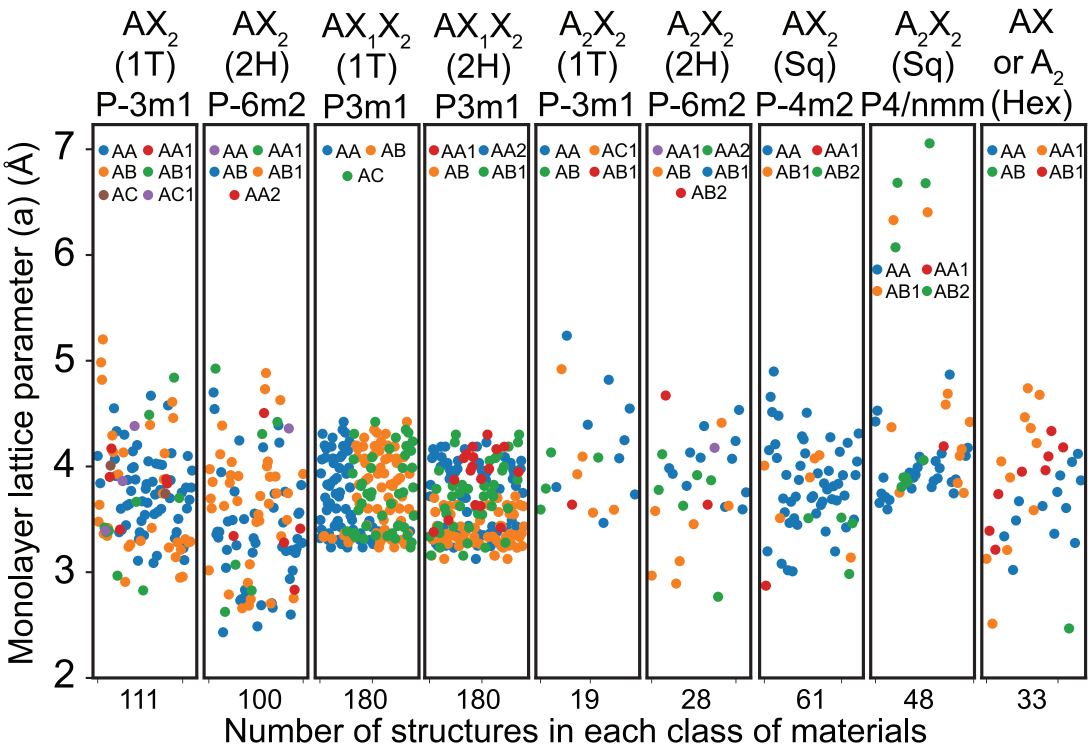

Figure S2: Monolayer lattice constant a (Å) as a function of number of materials for each monolayer class. The stacking patterns notation and color coding are the same as in Figure 2 of the main text for easier comparison with the lattice constant data for the bilayrs.

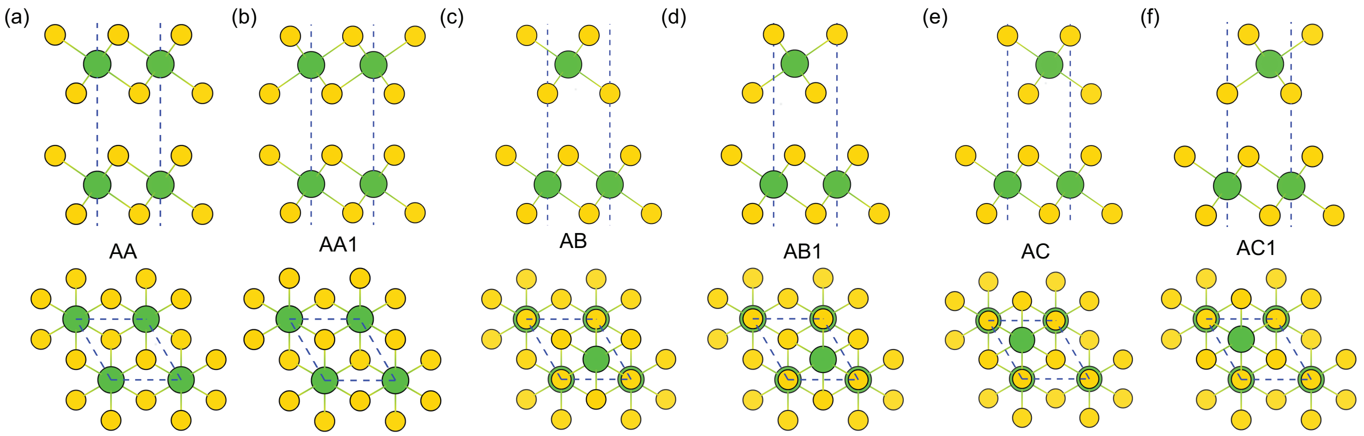

Figure S3: Stackings configurations for 1T-AX_2 hexagonal monolayers separated by a distance d to form the bilayers: (a) AA - the unit cells of each monolayer with the same in-plane orientation; (b) AA1 - the top monolayer unit cell of the AA stacking pattern is rotated by 180° with respect to the bottom layer; (c) AB and (d) AB1 - the top monolayer unit cell of the AA and AA1 stacking pattern are translated by $(2a/3,\ a/3)$ with respect to the bottom layer, respectively; (e) AC and (f) AC1 - the top monolayer unit cell of the AA and AA1 stacking pattern are translated by $(a/3,\ 2a/3)$ with respect to the bottom layer, respectively. Here a is the monolayer lattice constant.

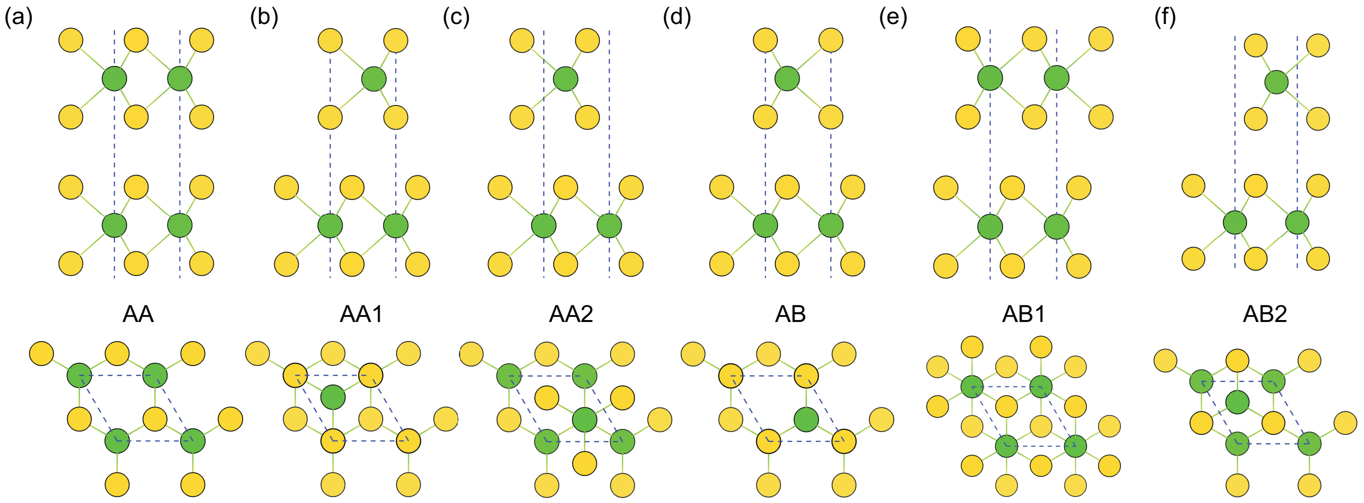

Figure S4: Stackings configurations for 2H-AX_2 hexagonal monolayers separated by a distance d to form the bilayers: (a) AA - the unit cells of each monolayer with the same in-plane orientation; (b) AA1 and (c) AA2 - the top monolayer unit cell of the AA stacking pattern is translated by ($a/3$, $2a/3$) and ($2a/3$, $a/3$) with respect to the bottom layer, respectively; (d) AB - the top monolayer unit cell of the AA stacking pattern is rotated by 180° with respect to the center of the A-X bond of the unit cell of the bottom layer; (e) AB1 and (f) AB2 - the top monolayer unit cell of the AB stacking pattern is translated by ($a/3$, $2a/3$) and ($2a/3$, $a/3$) with respect to the bottom layer, respectively. Here a is the monolayer lattice constant.

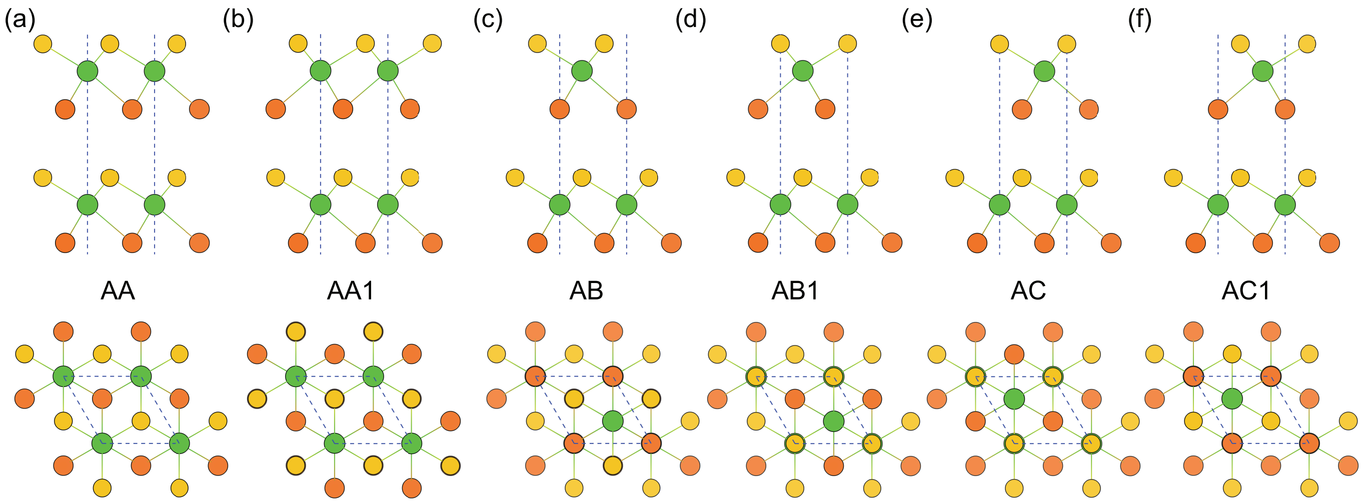

Figure S5: Stackings configurations for 1T-AX_1X_2 Janus monolayers separated by a distance d to form the bilayers with dissimilar atoms in the interface region: (a) AA - the unit cells of each monolayer with the same in-plane orientation; (b) AA1 - the top monolayer unit cell of the AA stacking pattern is rotated by 180° with respect to the bottom layer; (c) AB and (d) AB1 - the top monolayer unit cell of the AA and AA1 stacking pattern are translated by ($2a/3$, $a/3$) with respect to the bottom layer, respectively; similarly (e) AC and (f) AC1 - the top monolayer unit cell of the AA and AA1 stacking pattern are translated by ($a/3$, $2a/3$) with respect to the bottom layer, respectively. Note that the stacking configurations are the same as for the 1T-AX_2 monolayers shown in Figure S3 in which the same type of atoms are present in the interface region. Here a is the monolayer lattice constant.

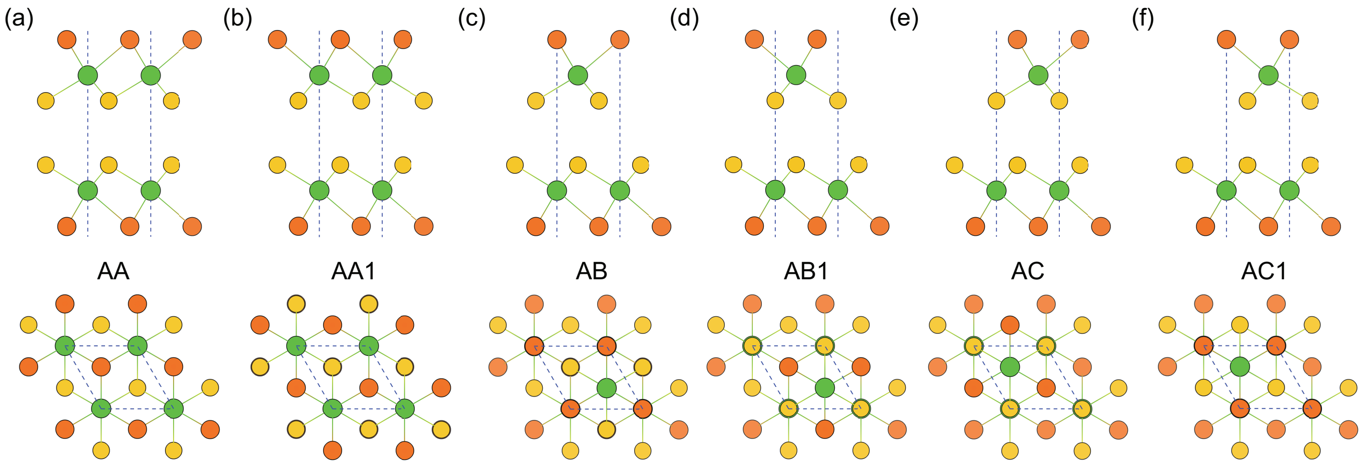

Figure S6: Stackings configurations for 1T-AX_1X_2 Janus monolayers separated by a distance d to form the bilayers with X_2-atoms in the interface region: (a) AA - the unit cells of each monolayer with the same in-plane orientation; (b) AA1 - the top monolayer unit cell of the AA stacking pattern is rotated by 180° with respect to the bottom layer; (c) AB and (d) AB1 - the top monolayer unit cell of the AA and AA1 stacking pattern are translated by ($2a/3$, $a/3$) with respect to the bottom layer, respectively; similarly (e) AC and (f) AC1 - the top monolayer unit cell of the AA and AA1 stacking pattern are translated by ($a/3$, $2a/3$) with respect to the bottom layer, respectively. Note that the stacking configurations are the same as for the 1T-AX_2 monolayers in Figure S3) in which the same type of atoms are present in the interface region. Here a is the monolayer lattice constant.

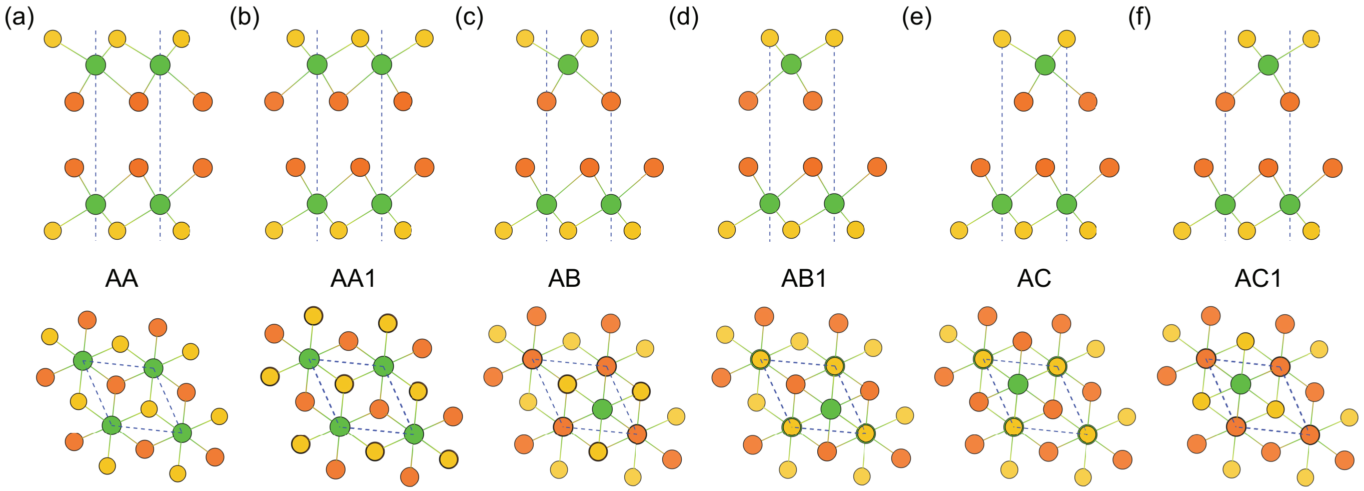

Figure S7: Stackings configurations for 1T-AX_1X_2 Janus monolayers separated by a distance d to form the bilayers with X_2-atoms in the interface region: (a) AA - the unit cells of each monolayer with the same in-plane orientation; (b) AA1 - the top monolayer unit cell of the AA stacking pattern is rotated by 180° with respect to the bottom layer; (c) AB and (d) AB1 - the top monolayer unit cell of the AA and AA1 stacking pattern are translated by ($2a/3$, $a/3$) with respect to the bottom layer, respectively; similarly (e) AC and (f) AC1 - the top monolayer unit cell of the AA and AA1 stacking pattern are translated by ($a/3$, $2a/3$) with respect to the bottom layer, respectively. Note that the stacking configurations are the same as for the 1T-AX_2 monolayers in Figure S3) in which the same type of atoms are present in the interface region. Here a is the monolayer lattice constant.

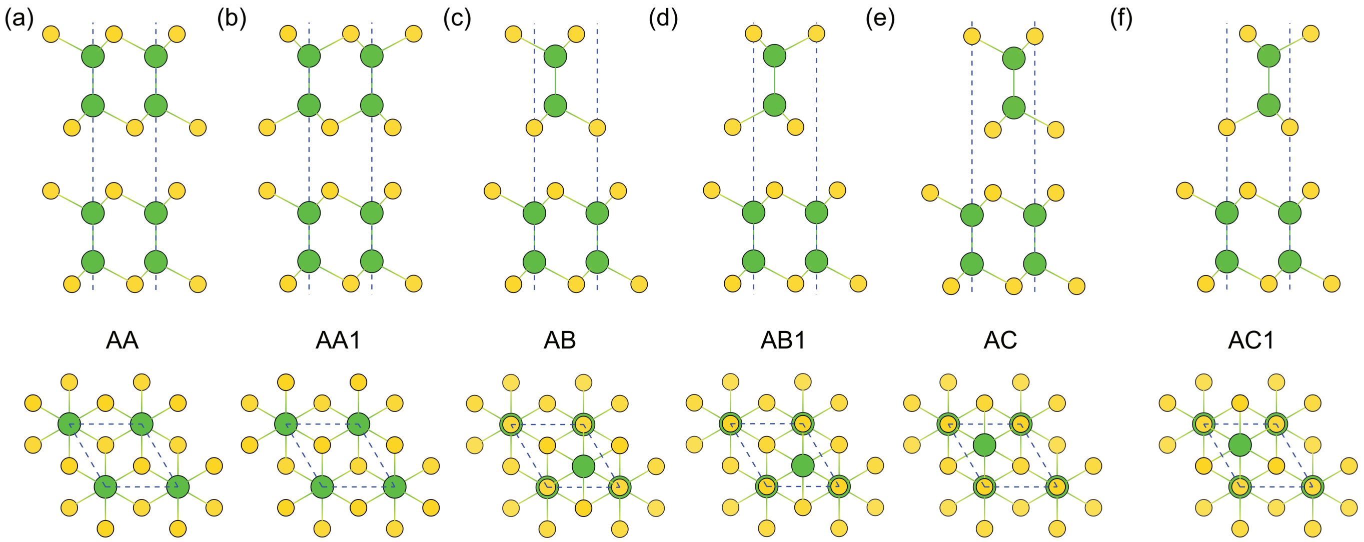

Figure S8: Stackings configurations for 1T-A_2X_2 hexagonal monolayers separated by a distance d to form the bilayers: (a) AA - the unit cells of each monolayer with the same in-plane orientation; (b) AA1 - the top monolayer unit cell of the AA stacking pattern is rotated by 180° with respect to the bottom layer; (c) AB and (d) AB1 - the top monolayer unit cell of the AA and AA1 stacking pattern are translated by $(2a/3,\ a/3)$ with respect to the bottom layer, respectively; similarly (e) AC and (f) AC1 - the top monolayer unit cell of the AA and AA1 stacking pattern are translated by $(a/3,\ 2a/3)$ with respect to the bottom layer, respectively. Note that the stacking configurations are the same as for the 1T-AX_2 monolayers in in Figure S3.Here a is the monolayer lattice constant.

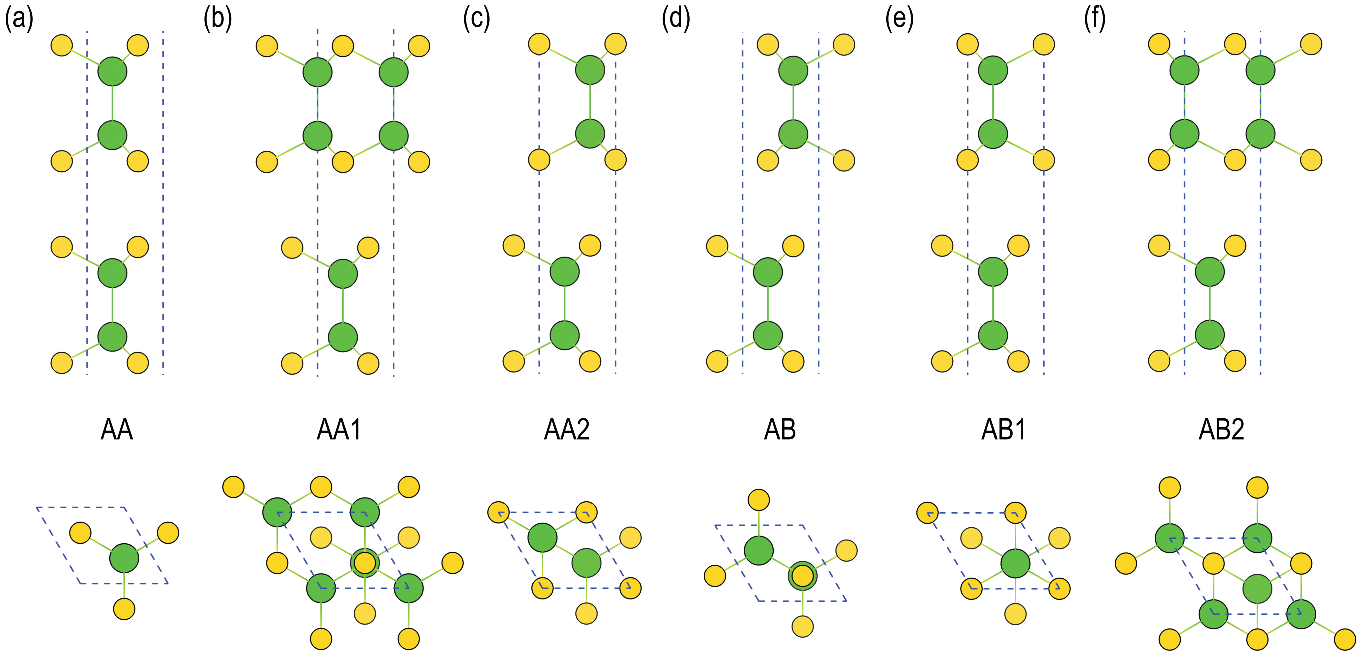

Figure S9: Stackings configurations for 2H-A_2X_2 hexagonal monolayers separated by a distance d to form the bilayers: (a) AA - the unit cells of each monolayer with the same in-plane orientation; (b) AA1 and (c) AA2 - the top monolayer unit cell of the AA stacking pattern is translated by $(a/3,\ 2a/3)$ and $(2a/3,\ a/3)$ with respect to the bottom layer, respectively; (d) AB - the top monolayer unit cell of the AA stacking pattern is rotated by 180° with respect to the center of the A-X bond of the unit cell of the bottom layer; (e) AB1 and (f) AB2 - the top monolayer unit cell of the AB stacking pattern is translated by $(a/3,\ 2a/3)$ and $(2a/3,\ a/3)$ with respect to the bottom layer, respectively. Note that the stacking configurations are the same as for the 2H-AX_2 monolayers in Figure S4. Here a is the monolayer lattice constant.

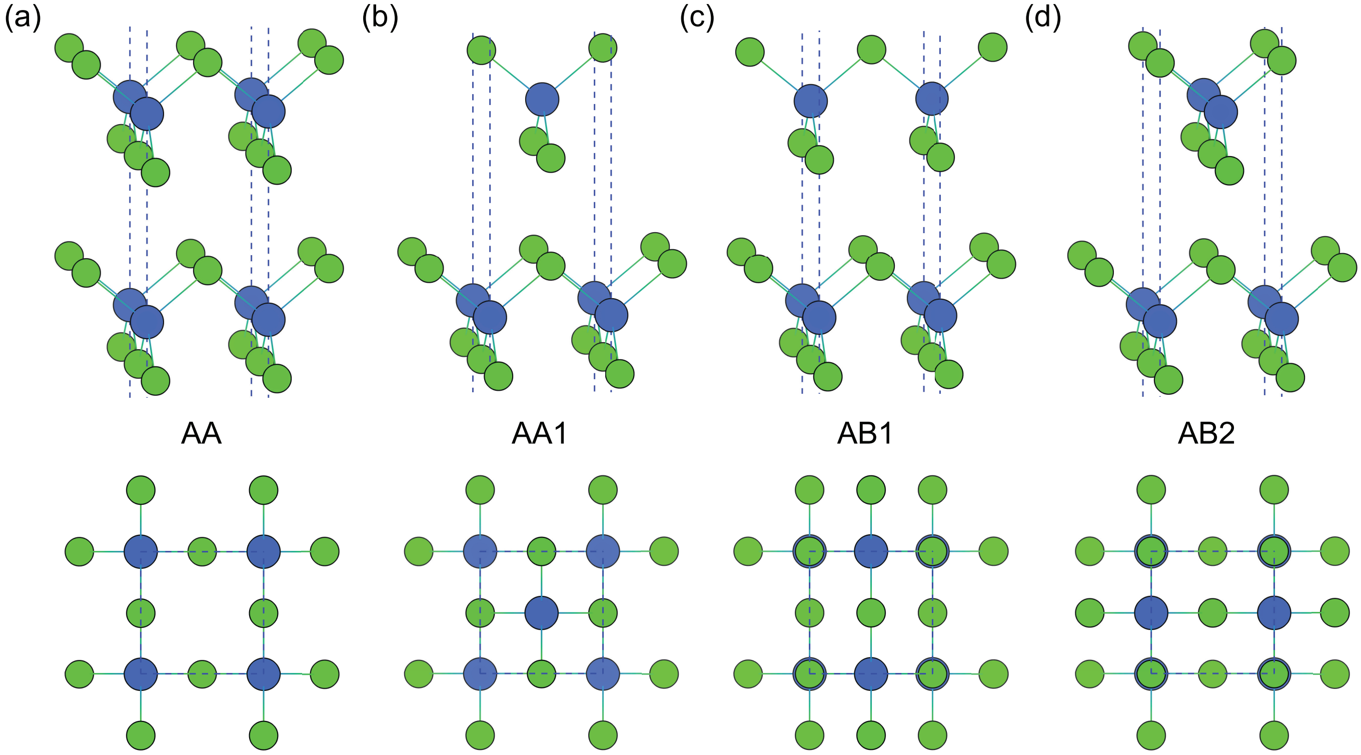

Figure S10: Stackings configurations for AX_2 square monolayers separated by a distance d to form the bilayers: (a) AA - the unit cells of each monolayer with the same in-plane orientation; (b) AA1 - the top monolayer unit cell of the AA stacking pattern is translated by $(a/2,\ a/2)$ with respect to the bottom layer; (c) AB1 - the top monolayer unit cell of the AA stacking pattern is translated by $(a/2,\ 0)$ with respect to the bottom layer; (d) AB2 - the top monolayer unit cell of the AA stacking pattern is translated by $(0,\ a/2)$ with respect to the bottom layer. Here a is the monolayer lattice constant.

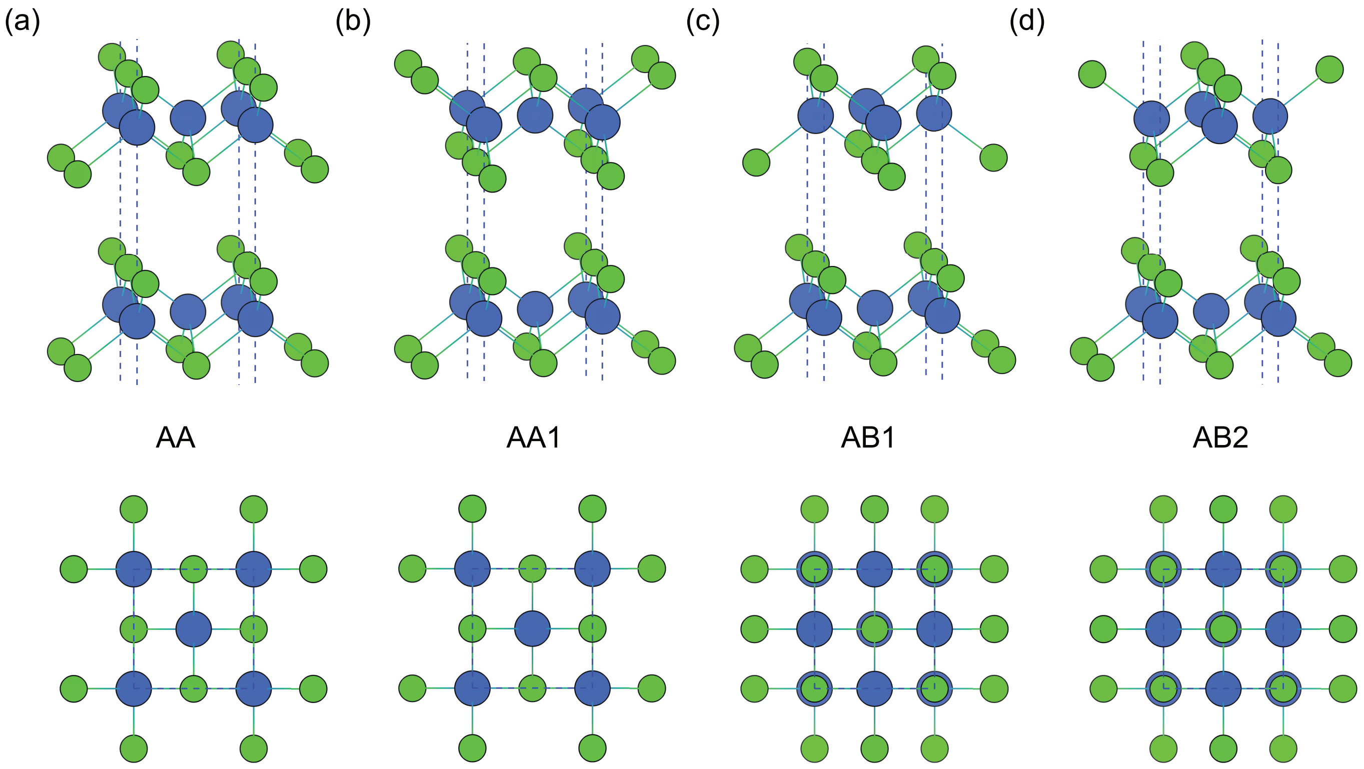

Figure S11: Stackings configurations for A_2X_2 square monolayers separated by a distance d to form the bilayers: (a) AA - the unit cells of each monolayer with the same in-plane orientation; (b) AA1 - the top monolayer unit cell of the AA stacking pattern is translated by $(a/2,\ a/2)$ with respect to the bottom layer; (c) AB1 - the top monolayer unit cell of the AA stacking pattern is translated by $(a/2,\ 0)$ with respect to the bottom layer; (d) AB2 - the top monolayer unit cell of the AA stacking pattern is translated by $(0,\ a/2)$ with respect to the bottom layer. Here a is the monolayer lattice constant. Note that the stacking configurations are the same as for the AX_2 square monolayers in Figure S10). Here a is the monolayer lattice constant.

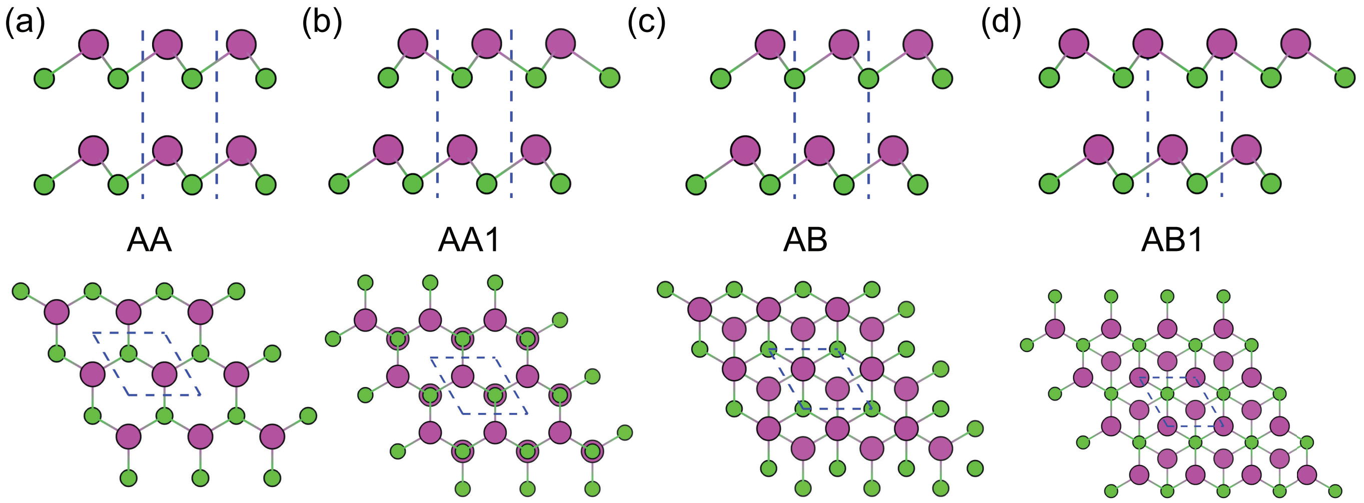

Figure S12: Stackings configurations for monolayers having two atoms (AX) in the unit cell separated by a distance d to form the bilayers: (a) AA - the unit cells of each monolayer with the same in-plane orientation; (b) AA1 - the top monolayer unit cell of the AA stacking pattern is rotated by 180° with respect to the bottom layer; (c) AB and (d) AB1 - the top monolayer unit cell of the AA and AA1 stacking pattern is translated by $(2a/3,\ a/3)$ with respect to the bottom layer, repectively. Note that graphene also belongs in this class, however, due to its planar structure only AA and AB are distinct configurations. Here a is the monolayer lattice constant.
